# Uncertainties in forces extracted from non-contact atomic force microscopy measurements by fitting of long-range background forces

**DOI:** 10.3762/bjnano.5.45

**Published:** 2014-04-01

**Authors:** Adam Sweetman, Andrew Stannard

**Affiliations:** 1 The School of Physics and Astronomy, The University of Nottingham, Nottingham, NG7 2RD, U.K.

**Keywords:** background subtraction, DFM, F(z), force, atomic resolution, NC-AFM, Si(111), STM, van der Waals

## Abstract

In principle, non-contact atomic force microscopy (NC-AFM) now readily allows for the measurement of forces with sub-nanonewton precision on the atomic scale. In practice, however, the extraction of the often desired ‘short-range’ force from the experimental observable (frequency shift) is often far from trivial. In most cases there is a significant contribution to the total tip–sample force due to non-site-specific van der Waals and electrostatic forces. Typically, the contribution from these forces must be removed before the results of the experiment can be successfully interpreted, often by comparison to density functional theory calculations. In this paper we compare the ‘on-minus-off’ method for extracting site-specific forces to a commonly used extrapolation method modelling the long-range forces using a simple power law. By examining the behaviour of the fitting method in the case of two radically different interaction potentials we show that significant uncertainties in the final extracted forces may result from use of the extrapolation method.

## Introduction

Non-contact atomic force microscopy (NC-AFM) is now the tool of choice for surface scientists wishing to investigate interatomic and intermolecular forces on surfaces with sub-Angstrom precision. Although in principle it is relatively straightforward to extract the tip–sample force from the experimental observable (i.e., the shift in the resonant frequency of the oscillating cantilever Δ*f*), in practice a significant amount of processing is usually required in order to obtain the desired quantity.

In this paper the focus primarily concerns the imaging and quantitative interpretation of atomic or molecular resolution NC-AFM experiments conducted in ultrahigh vacuum (UHV). In these experiments, the quantity of interest is usually the site-specific/short-range force between the very apex of the tip and the surface. In any atomic resolution experiment using a scanning probe, atomic contrast must arise from an interaction that decays on a distance comparable to the interatomic spacing, otherwise atomic resolution would not be readily obtained. Consequently, the tip–sample interaction is usually modelled (for example using density functional theory (DFT) [1]) as the interaction between a small cluster of atoms (representing the tip) and a slab of surface atoms.

In order to extract the short-range force from the frequency shift measurement, however, the contribution from non-site-specific (i.e., long-range) forces must be removed. These are normally van der Waals and electrostatic in origin (here we ignore more complex cases such as magnetic systems).

The ‘gold standard’ for performing this subtraction is the so-called ‘on-minus-off’ method utilised by Lantz et al. [2], and Ternes et al. [3], amongst others. The principle behind this subtraction is quite simple: if there exists a region on the surface that is otherwise identical to the position at which the short-range force is to be measured, but is missing the atom or molecule that produces the short-range interaction, then performing the same measurement over that region will provide a measurement containing only the contribution of the long-range forces. A simple case is that of an adsorbed atom or molecule on a surface.

**Figure 1:**
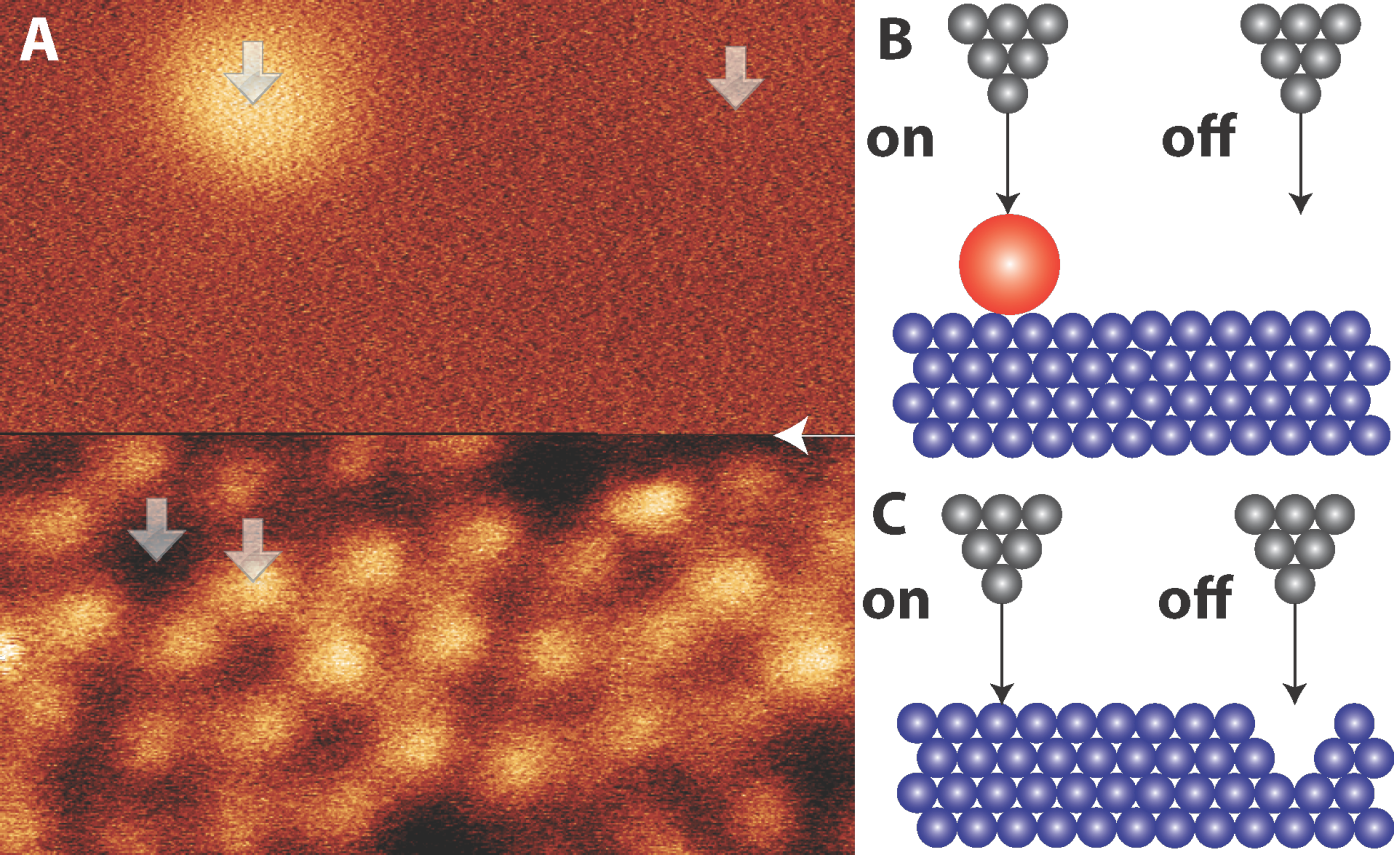
A) Constant Δ*f* NC-AFM image of a C_60_ molecule adsorbed on the Si(111)-(7 × 7) surface showing atomic and molecular resolution. The position of the white arrow shows where the Δ*f* setpoint was changed from Δ*f* = −53 Hz (adatoms, lower half of image) to Δ*f* = −26.5 Hz (C_60_, upper half of image). Larger arrows show the Δ*f*(*z*) spectra positions. *V_gap_* = 0 V. *A_0_* = 0.11 nm. *f*_0_ = 24866.3 Hz. B) and C) Cartoon representations showing the principle behind ‘on-minus-off’ measurements on a molecule and surface adatom respectively.

A measurement is first performed over the molecule, the tip is then moved some distance to the side and another measurement is performed over the same range of tip–sample separations. The contribution to the total force from the interaction between the macroscopic part of the tip and the bulk surface is the same, but the contribution from the molecule is removed. A similar procedure can be utilised for surface atoms if there is a large enough ‘empty’ region on a flat surface that does not exert any short-range force. A well-known example of this is the cornerhole on the Si(111)-(7 × 7) surface [2]. A cartoon of these two cases is shown in Figure 1B and Figure 1C.

Although the ‘on-minus-off’ technique provides a conceptually simple way of removing the long-range contribution, it has the limitation that it can only be applied on surfaces where such ‘null sites’ exist. In practice, on the vast majority of clean well-reconstructed surfaces, no such sites are available. In these instances attempts have been made to remove the long-range contribution by fitting the long-range background to a series of inverse power laws [4], and extrapolating the long-range force behaviour into the region where the short-range contributions are present. Although it is true that the long-range dispersion and electrostatic contributions might in principle be approximated by equations of this type, there has been surprisingly little discussion in the literature as to the uncertainties introduced using this technique. It is trivially true that any form of extrapolation must introduce a degree of uncertainty, but beyond this, there has been very little discussion regarding the uncertainties introduced during application of this technique to real experimental data, although some authors have provided estimates [5–6], or explicitly chosen not to utilise the technique [7]. A notable exception to this is the discussion that has surrounded Kelvin probe force microscopy (KPFM) where accurate modelling of this long-range regime is critical to interpreting results [8–10]. Nonetheless, long-range forces are readily subtracted in the literature using this method, often using simplistic models [1,6,11–14]. Results are then often compared to DFT modelling with subsequent interpretation of the data requiring accuracies on the order of a few 100’s [1,13], or sometimes even 10’s [12], of piconewtons. Interestingly, this technique has sometimes been applied in instances where ‘off’ measurements are, in principle, available [6,11].

In this paper we perform a simple set of force measurements using the same tip apex on two different surface locations where we are able to use the ‘on-minus-off’ method. This is done by depositing C_60_ molecules onto a clean Si(111)-(7 × 7) surface, and subsequently examining the both the tip–C_60_ and tip-silicon interactions. This method provides a useful way of checking the validity of the fitting method as we have access to two different interaction potentials (with ‘on’ and ‘off’ curves available in both cases), against which to test the long-range extrapolation method.

We find that although some fits do indeed recover similar force profiles to the ‘on-minus-off’ method, we show that there is no way of determining, *a priori*, which fit is correct without access to the ‘on-minus-off’ result. Consequently, we suggest that significant uncertainties may result from short-range forces extracted by this method on surfaces where no check is available.

## Methods

The data in this paper were acquired using an Omicron Nanotechnology GmbH combined LT-STM/NC-AFM operating in UHV and at cryogenic temperatures (78 K at LN_2_). Clean Si(111)-(7 × 7) samples were prepared by standard flash annealing to 1200 °C, rapid cooling to 900 °C, and then slow cooling to room temperature. A low coverage of C_60_ was prepared by depositing the molecules from a tantalum pocket onto the room temperature substrate. Following deposition the sample was immediately transferred into the scan head and left to cool before imaging.

Commercial qPlus sensors from Omicron with electrochemically etched tungsten wire glued to one tine of the tuning fork were introduced into the scan head without any further preparation. We typically recorded resonant frequencies of *f*_0_ ≈ 25 kHz, and, based on previous measurements of similar sensors [5,15], assume an effective stiffness of *k* ≈ 2000 N/m. The sensors were first prepared on a clean silicon surface by standard STM techniques (pulsing and indentation) until good STM and NC-AFM resolution was achieved. Typically we used oscillation amplitudes (*A*_0_) of between 0.1 and 0.3 nm during NC-AFM imaging. In order to eliminate any possible effect from either electronic crosstalk [16] or the so-called “Phantom Force” [17] all NC-AFM imaging was performed at 0 V (i.e., no detectable tunnel current). To stabilise the imaging conditions a custom-built atom tracking system developed at the University of Mainz [18] was used to apply feedforward correction to reduce the effect of thermal drift and piezo-electric creep.

To obtain the site-specific interaction force, single point Δ*f*(*z*) spectroscopy measurements were acquired on the adatoms, the cornerholes, the molecules, and ‘off’ the molecules, with all the spectra having identical parameters. In order to eliminate artefacts in the subtraction due to the shift in height due to the topographic feedback, the 'on' spectra were first aligned (on the *z* axis) to the 'off' spectra by a least mean squares fitting to the long-range part of the interaction [19] (this gave the same alignment within error as the method described by Sugimoto et al. [20]). The ‘off’ curve was then subtracted from the ‘on’ spectra and the resultant short-range Δ*f*(*z*) was inverted to force using the Sader–Jarvis formula [21]. Full technical details of the force extraction procedure, including the implementation of the force inversion algorithm and alignment procedure used for the ‘on-minus-off’ measurements, are presented in a forthcoming publication [19]. All data presented is the result of single Δ*f*(*z*) measurements and no averaging of curves has been performed to improve the signal-to-noise ratio.

In general, in order to perform long-range background subtraction, short-range curves are acquired and then aligned with a separate long-range curve before fitting, which can introduce additional uncertainties. In order to make a fairer comparison we performed high data density spectra out to long-range in all four positions. This ensured that the alignment of the ‘on’ and ‘off’ curves was identical for both the ‘on-minus-off’ method and the long-range extrapolation method.

We used a simple power law of form *a*/(*z* + *b*)*^c^* + *d* to fit the long-range part of the curve (using the standard curve fitting toolbox in MATLAB), assuming the tip–surface configuration can be modelled as a simple geometric shape positioned above a plane. Here *a* is related to the Hamaker constant of the material and size of the tip, *b* describes the divergence point of the long-range forces, *c* is the exponent governing the decay of the force, and *d* is an offset term taking into account any small deviation of the Δ*f*(*z*) tail from zero.

Although this form is almost certainly an oversimplification of the real interaction, it has been commonly applied [1,6,11–14] in these types of experiment. We note in passing that even for this simple function it was necessary to constrain the range and starting value of the fit parameters in order to ensure reliable convergence of the curve fitting algorithm (for example the parameter *c* was usually constrained to be between 1 and 3). All parameters were allowed to fully relax within the constraints that allowed for reliable convergence of the curve fitting algorithm, and we note that none of the fit parameter values were limited at the constraint boundaries for any of the fits presented here. In this work we did not investigate the effect on the fit due to the constraining or limiting of the free fit parameters, instead only analysing the fit that gave the best residuals for a given exclusion point (see below) for a full relaxation of all the fit parameters.

A key parameter in the curve fitting (not explicit in the equation itself) is how much of the curve to fit, as fitting part of the curve where short-range interactions are present will distort the form of the resultant fit, which should only approximate the long-range dispersion interactions. Although there is no definitive solution to determining where the short-range forces ‘turn on’, an estimate can be made by examining the Δ*f* spectra taken over different sites. The point in *z* where the curves start to diverge can be taken as an estimate for the point where the measurement starts to become sensitive to site-specific interactions.

## Results

Figure 1A shows a constant Δ*f* image of a C_60_ molecule adsorbed on the Si(111)-(7 × 7) surface. In order to obtain atomic resolution on the substrate, and image the molecule without perturbing it [15,22], the setpoint was changed halfway up the image (see figure caption). In this instance the molecule is imaged at a low setpoint to reduce the chance of perturbing the tip state, and consequently no sub-molecular resolution is obtained. After obtaining the image, single point Δ*f*(*z*) spectra were taken on the silicon adatoms, the cornerholes, on top of the molecule, and ‘off’ the molecule.

**Figure 2:**
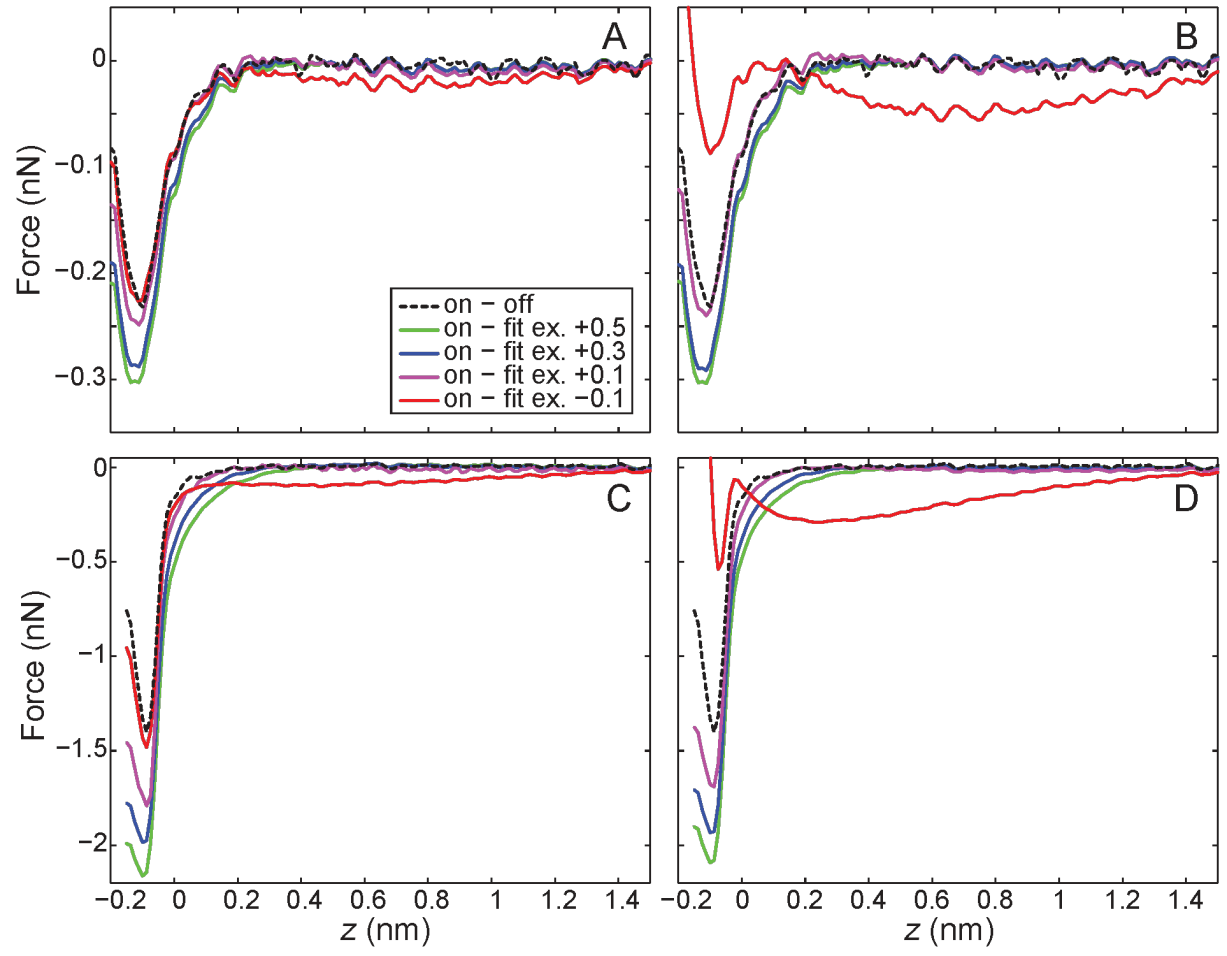
Extracted short-range force curves from ‘on-minus-off’ extraction, and comparison to long-range fitting for A) Tip–C_60_ interaction fitting to ‘off’ curve, B) Tip–C_60_ interaction fitting to ‘on’ curve, C) Tip–Si interaction fitting to ‘off’ curve, D) Tip–Si interaction fitting to ‘on’ curve. In the legend ‘ex’ indicates the point below which data was excluded from the fitting (e.g., ex = +0.1 indicates any data below +0.1 nm was excluded from the fit).

Short-range forces were extracted by the two methods described in the experimental section. First by the ‘on-minus-off’ method, second by extrapolating a fit of the long-range force into the short-range regime. To test the consistency of the extrapolation method we produced fits using the same fitting method for both the ‘on’ and ‘off’ curves (noting that in an experiment requiring long-range extrapolation only the ‘on’ curve is available); i.e., fitting the long-range part of the curve using the power law described in the methods section, excluding different amounts of the short-range data and monitoring the subsequent effect on the extracted short-range forces. The resultant short-range forces, extracted by both methods, for the tip–sample interaction over both the silicon adatoms and the C_60_ molecule are shown in Figure 2.

Examining first the results on the C_60_ molecule, the ‘on-minus-off’ method shows a weak attractive force between tip and sample, suggesting either a molecular or weakly interacting silicon tip apex [23] which does not form a strong covalent bond with the molecule. Examining the short-range forces extracted by long-range extrapolation, fitting to the 'off' curve (Figure 2A), it is clear that the two fits excluding data below 0.5 and 0.3 nm systematically overestimate the short-range force, whereas the fit excluding ≤0.1 nm recovers a profile very close to the ‘on-minus-off’ method. Although the fit excluding ≤−0.1 nm obtains a more accurate minimum force value, we note the deviations in the tail show that the power law does not produce a good fit, and this is also clear in the residuals produced during curve fitting. Fitting to the ‘on’ curve produces similar results, except that the deviation in the fit when fitting down to −0.1 nm is much more pronounced, as we are clearly attempting to fit part of the short-range interaction, present in the on curve, using the power law.

With respect to the tip–silicon results (Figure 2C and Figure 2D), the force profiles from ‘on-minus-off’ are consistent with chemical bond formation between the tip apex and the reactive silicon adatom. Turning to the results obtained by long-range extrapolation, we observe a similar relative behaviour between the different fits as for the C_60_ results, with the notable exception that none of the curves accurately recover the correct short-range force profile, as all of the curves systematically overestimate the total short-range force, or show deviations due to failure of the power law fit.

**Figure 3:**
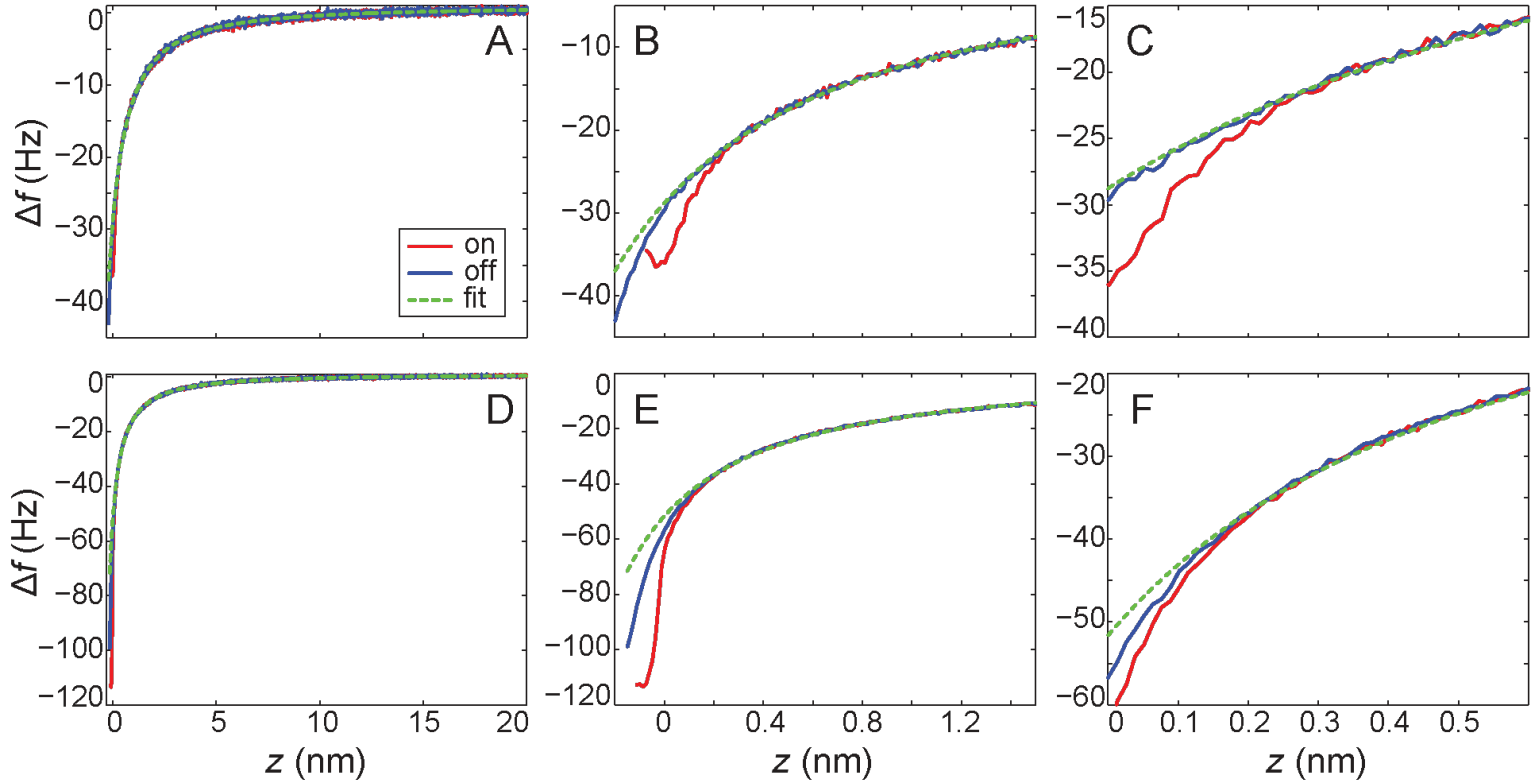
Close inspection of the divergence point between the ‘on’ and ‘off’ curves for A)–C) tip–C_60_ interaction, and D)–F) tip–Si interaction. Also plotted is the long-range fit for a cut-off of +0.1 nm which resulted in the short-range forces plotted in Figure 2. A)–C) shows the same data plotted on three different axis scale to show A) the long-range behaviour of the fit, B) the behaviour in the short-range regime, and C) the divergence point of the ‘on’ and ‘off’ curves. D)–E) shows the same progression for the tip–Si interaction.

An important subtlety here is the choice of the exclusion position, or rather, exactly how the exclusion position is determined for a given dataset. Although on initial examination of the force curves it might be assumed that the fit excluding ≤0.1 nm provides a reasonable approximation to the ‘on-minus-off’ method, if we examine the raw Δ*f* curves in detail (Figure 3A–C for the C_60_ data, D–F for Si data) it is interesting to note that if the ‘on-minus-off’ curve was not available for comparison we would have no reason to select this as the correct cut-off position. The divergence of the curves occurs somewhere between 0.2 nm and 0.3 nm, which should, in principle, strongly guide the choice of cut-off that determines which data to exclude from the fit. Therefore the fit excluding ≤0.1 nm actually fits part of the short-range interaction, and its agreement with the ‘on-minus-off’ method is purely fortuitous.

Consequently, in the absence of the ‘on-minus-off’ method as a check, the most rigorous position at which to start excluding data would be at approximately 0.3 nm. If this position were used, the overestimation of the short-range force would be approximately 20% in the case of the tip–C_60_ interaction, and approximately 40% in the case of the tip–silicon adatom interaction. Importantly, we note that these force values are all within the ‘sensible’ range of forces that might be expected for different tip structures common in this type of experiment. As such, if the forces were extracted using this method in an instance where no ‘on-minus-off’ check were possible, there would be no obvious reason to doubt their accuracy, especially if there was fortuitous agreement with results obtained from modelling calculations. In particular it is important to note that these uncertainties are larger than the systematic uncertainties usually present in NC-AFM experiments (usually dominated by the uncertainty in the oscillation amplitude of the cantilever), and critically, there is no reason to expect that the trend in the fit would to be systematic from tip to tip.

It is this uncertainty that lies at the crux of the matter regarding long-range background extrapolation methods. We wish to stress that it is not the case that the extraction of forces in this manner necessarily produces incorrect, or unphysical, results, or even that the technique cannot in principle provide the ‘correct’ result. The issue is that in the absence of any independent check it is extremely difficult to quantify the uncertainty in the final extracted quantities. We again stress that the model used here to fit the long-range background is, although commonly used, an oversimplification, and a valid argument could be made that a more complex model, taking into account more details of the tip geometry, would be more robust.

In principle it is clear that more realistic models should better reflect the physical reality of the system, but an inherent issue is that these models introduce an even larger number of free parameters into the fit. Even if these parameters are weakly constrained to use ‘physical’ parameters, the range of possible fits (all producing ‘good’ fits to the long-range data) grows dramatically as the number of free parameters is increased. Most importantly, the fact that a given function produces a ‘good’ fit to the selected range of data does not, in itself, provide strong evidence that the extrapolation into the short-range force regime is accurate.

We note that the confidence in the fit to the long-range behaviour may be increased dramatically if a judicious knowledge of the tip structure is available, for example by use of *in situ* field ion microscopy (FIM), transmission electron microscopy (TEM), and/or scanning electron microscopy (SEM), on well-defined tips both before and after force spectroscopy experiments have been performed. If used on tips made from a single, well-characterised material, such methods might provide extremely strong bounds with which to constrain the free parameters of the fit, and the choice of tip model to be used. Consequently, we expect the uncertainties introduced from the fit could be reduced, and well-quantified, in such instances.

Although these techniques are sometimes used [24], in the vast majority of experimental setups these facilities are not available, and, even if available, drastically increase the time and difficulty in performing the measurements, as any indentation of the tip into the surface will require the tip structure checks to be repeated. This is likely to be even more important in the case of experiments using qPlus-type setups, where STM tip treatment methods are often used to prepare tips *in situ* on the surface. In these cases, significant transfer of material from tip to surface, and vice versa, can occur, and dramatically modify the long-range background profile.

Consequently, we suggest as a practical guide that ‘site-difference’ measurements, where the difference between two ‘on’ curves is taken [7,25], are used to make comparisons to calculated results on surfaces where ‘on-minus-off’ experiments are not feasible, or, if the absolute short-range force must be extracted by the extrapolation method, a discussion of the uncertainties should be presented. An estimate of the errors might be obtained practically by obtaining a number of fits with different models/parameters, and systematically varying the cut-off position of the fits. If the curve fitting algorithm is robust under different constraints and starting parameters, and different models return similar physical properties of the tip, then it seems that a robust estimate of the resultant uncertainties might be made.

## Conclusion

In conclusion, we have presented a comparison of the results obtained from extracting site-specific forces in NC-AFM by ‘on-minus-off’ and extrapolation methods. Although extrapolation techniques can provide accurate force values, a significant uncertainty is introduced into the quantitative values of the resulting short-range forces. We recommend that the ‘on-minus-off’ technique is used where possible, and a judicious consideration of the uncertainties is presented when extrapolation techniques must be used, especially when comparing the results to calculated values. We also note that during the review process we became aware of a forthcoming publication by Kuhn et al. [26] which rigorously explores the uncertainties and consistency of the long-range background fitting method for a number of different tip–surface interaction models in the case where no ‘off’ curve is available, using a conventional silicon cantilever NC-AFM setup.
